# The Commitment to Excellence: Understanding Nurses’ Perspectives on Continuous Professional Development

**DOI:** 10.3390/healthcare12030379

**Published:** 2024-02-01

**Authors:** Biljana Kurtović, Petra Gulić, Snježana Čukljek, Biserka Sedić, Martina Smrekar, Sanja Ledinski Fičko

**Affiliations:** 1 Department of Nursing, University of Applied Health Sciences, 10000 Zagreb, Croatia; snjezana.cukljek@zvu.hr (S.Č.); biserka.sedic@zvu.hr (B.S.); martina.smrekar@zvu.hr (M.S.); sanja.ledinski-ficko@zvu.hr (S.L.F.); 2 Department of Nursing, Faculty of Health Studies, University of Rijeka, 51000 Rijeka, Croatia; 3 Department of Emergency, Intensive Medicine, and Clinical Pharmacology with Toxicology, Clinic for Internal Diseases, University Hospital Centre Split, 21000 Split, Croatia; petra.gulic418@gmail.com

**Keywords:** continuous professional development, nurse motivation, healthcare education, nursing workforce

## Abstract

Continuous Professional Development (CPD) is essential for nurses to maintain up-to-date knowledge and skills in the evolving healthcare sector. This study explores nurses’ attitudes toward CPD, its necessity, and the challenges encountered. The aim is to examine nurses’ perspectives on CPD, focusing on their participation and motivation, in relation to their workplace, workplace function, and form of work. A cross-sectional study design was employed with 151 nurses from University Hospital Centre Split, Croatia. Data were collected using the “Professional Development of Nurses questionnaire (Q-PDN)” and analyzed using descriptive statistical methods, the Kolmogorov–Smirnov test, Pearson’s correlation, ANOVA test, and *t*-test. The average level of participation in CPD activities was 4.27 (±0.63), indicating a positive inclination towards CPD. The study identified a statistically significant difference in activities related to CPD (t = 2.12; *p* = 0.036) among employees of surgical and intensive care units compared to other departments, where a higher level of engagement was present among employees of other departments. Notably, nurses without managerial roles showed 0.16 points higher participation in CPD activities compared to their managerial counterparts, though this was not statistically significant (t = 0.92; *p* = 0.357). Nurses in managerial roles valued CPD for professional development more highly, with a significant difference (t = 2.77; *p* = 0.006). Full-time nurses demonstrated a higher perception of the importance of personal professional development compared to part-time nurses, with a significant difference (F = 2.88; *p* = 0.038). The study reveals a strong commitment to CPD among nurses, with variations based on workplace roles and schedules. It underscores the need for role-specific and adaptable CPD programs to meet diverse needs and enhance professional competence in the nursing workforce.

## 1. Introduction

The commitment to excellence in nursing is intrinsically linked to Continuous Professional Development (CPD), a central aspect of nurses’ lifelong learning. CPD is vital for maintaining up-to-date knowledge and skills essential in the ever-evolving healthcare sector. Understanding nurses’ perspectives on CPD is crucial as it directly affects patient care quality, safety, nurse satisfaction, and healthcare costs [1,2,3]. Furthermore, well-structured CPD programs play a pivotal role in refining patient-centered care practices, ensuring that nurses are adeptly equipped to address the unique and changing needs of patients within a rapidly transforming healthcare environment [4].

CPD is not just a requirement; it is a core value that shapes nursing professionalism. It extends the practitioner’s ability beyond initial training and qualifications, contributing to competent practice and high standards of nursing care. This commitment to professionalism and lifelong learning is reflected in nurses’ attitudes and motivation as they view CPD as essential for keeping their knowledge and skills current and improving patient care standards [5].

The interplay between CPD and nursing professionalism manifests in how CPD initiatives directly influence the quality of nursing practices and patient outcomes. The dynamic nature of the healthcare sector necessitates a continual update of skills and knowledge, where CPD serves as a bridge between evolving healthcare demands and nurses’ ability to meet these challenges effectively.

Recent studies have revealed key themes in nurses’ experiences with CPD. These include the influence of organizational culture, the necessity of supportive environments, and the importance of attitudes and motivation reflecting professional values [6,7,8]. Additionally, nurses perceive certain barriers to CPD such as staffing levels, workload, and funding issues. Importantly, the impact of CPD on practice is both direct and indirect, depending on organizational support and culture [5].

Despite its recognized importance, CPD does not always address the real needs of nurses. There are variations in CPD requirements across different countries, with some mandating it while others leave it to the nurses’ discretion. This discrepancy can lead to uneven engagement in CPD activities. Furthermore, the studies highlight the need for CPD programs to be more attainable, realistic, and relevant, with adequate funding and accessibility [9,10]. This underscores the global diversity in CPD approaches, not only in terms of mandatory requirements but also in its perception and implementation across various healthcare systems. The need for a comprehensive and varied understanding of CPD in nursing is thereby accentuated, advocating for CPD frameworks that are adaptable to diverse nursing contexts and roles [11].

Upon completion of formal education and obtaining a nursing license, nurses have the right and obligation to continuously enhance their acquired knowledge. This involves supplementing and adopting new knowledge and specific skills in line with the latest advancements and insights in the field of nursing necessary for adequate healthcare [12]. In Croatia, CPD for nurses is regulated under specific guidelines set by the Croatian Nursing Council. The framework mandates that upon completion of formal education and obtaining a nursing license, nurses are obliged to continuously enhance their knowledge and skills. This involves engaging in various CPD activities, such as congresses, symposia, courses, and lectures, that align with the latest advancements and insights in the field of nursing, which are crucial for delivering adequate healthcare. To maintain and renew their nursing license, Croatian nurses must accumulate a certain number of CPD points. Specifically, they are required to collect 15 CPD points annually, culminating in a total of 90 points over a six-year period. Each CPD activity carries a certain amount of CPD points as prescribed by the “Regulation on the content, deadlines, and procedure for continuous professional development and verification of professional competence of nurses” [13]. This structured approach ensures that nurses remain updated with the evolving standards and practices in nursing care. The Croatian Nursing Council meticulously monitors this process, electronically recording the accumulation of CPD points for each nurse and issuing a new license upon the completion of the six-year period.

Although numerous studies [1,2,3,4,5] have found the need for ongoing professional development of nurses, insufficient knowledge exists about nurses’ perceptions of the activities and purposes of continuous professional development. Nurses’ attitudes may vary depending on a range of factors including organizational support, availability of resources, personal motivations, and perceptions of the benefits of ongoing development. Organizational resources, reward systems, and recognition of nurses’ roles in development processes can be significant factors shaping their motivation and commitment to this crucial aspect of professional development.

The commitment to excellence in nursing through CPD is a multifaceted issue. By understanding nurses’ perspectives and addressing the identified challenges, healthcare organizations can foster an environment conducive to professional growth, ultimately benefiting patient care and the nursing profession.

In Croatia, to date, no study has been conducted on nurses’ attitudes towards CPD across different hospital departments. By investigating these attitudes, particularly focusing on how different workplaces function, the form of work, and the specificity of the workplace influence nurses’ engagement in CPD, this study aims to fill this knowledge gap.

The aim of this study is to examine nurses’ attitudes towards CPD to determine participation and motivation for ongoing professional development concerning their workplace, workplace function, and form of work.

## 2. Materials and Methods

### 2.1. Study Design

This research was conducted utilizing a cross-sectional study design.

### 2.2. Participants

The study encompassed a cohort of 151 nurses from University Hospital Centre Split, Croatia. These participants were selected from various hospital nursing backgrounds to provide a diverse and comprehensive understanding of the topic.

### 2.3. Selection Criteria

The study included a diverse cohort of nursing professionals from the Clinical Hospital Centre Split, Croatia, based on the following criteria:

Inclusion criteria: general care nurses with a Bachelor of Nursing, Master’s in Nursing, or Ph.D. in Nursing; nurses involved in shift work, including different shifts and on-call duties; and nurses working across various units within the institution.

Exclusion criteria: non-nursing health professionals.

### 2.4. Procedure

The study was conducted at the Clinical Hospital Centre Split, involving nurses from various departments. The research methodology employed an anonymous online questionnaire to gather data, ensuring the confidentiality and safety of participant responses. The questionnaire was distributed via an internal link within the Clinical Hospital Centre Split. This method was chosen to efficiently reach a wide range of nursing professionals within the institution, ensuring a diverse representation of experiences and perspectives, and allowing nurses to conveniently participate without compromising the integrity of the research. This approach facilitated broad participation and streamlined data collection within the institution.

### 2.5. Instrument

We translated the “Professional Development of Nurses questionnaire (Q-PDN)”, developed by Brekelmans [14], into Croatian. The translation process was carried out by two independent professional translators to ensure linguistic accuracy. To ensure cultural relevance, the translated version was reviewed by a panel of Croatian nursing professionals. This step was crucial for confirming the cultural appropriateness and applicability of the questionnaire in the Croatian nursing context. After minor modifications based on the panel’s feedback, a pilot test was conducted with Croatian nurses to assess clarity and understandability. The reliability of the translated instrument was validated through Cronbach’s alpha, which indicated a high level of internal consistency (α = 0.95). The questionnaire is structured to assess nurses’ participation in professional development activities, the conditions they consider necessary for involvement, and their actual engagement in these activities. Responses were quantified using a Likert scale.

### 2.6. Analysis

The analysis was conducted using the STATISTICA 12 software package by Tibco, California, 2013. Data were analyzed using descriptive statistical methods, including the calculation of means and standard deviations. The normality of the distribution of responses was assessed using the Kolmogorov–Smirnov test. To explore the relationship between participation and motivation for continuous professional development with age, Pearson’s correlation was utilized. The ANOVA test was employed to examine differences in attitudes towards participation and motivation for continuous professional development, as well as professional development in relation to the level of education. The importance of professional development with respect to function was analyzed using the *T*-test. Additionally, the *T*-test was also used to determine differences in attitudes towards continuous professional development based on work location.

### 2.7. Ethics

The study was conducted with the approval of the University Hospital Centre’s ethical committee (Class: 500-03/23-01/05, Approval No. 2181-147/01/06/LJ.Z.-23-02). Informed consent was obtained from all participants prior to their involvement in the study. By signing the informed consent, participants acknowledged their willingness to take part in the research, asserting that their participation was entirely voluntary. Furthermore, they were assured of their right to withdraw from the study at any point without any repercussions. Permission to use the instrument was obtained directly from the author.

## 3. Results

Demographic characteristics of the participants are presented in Table 1.

Table 2 presents the extent of nurses’ engagement in CPD activities, highlighting factors crucial for the renewal of their licenses. The average level of participation in continuous professional development activities is 4.27 with a standard deviation of 0.63 from the mean.

Participation in CPD activities is predominantly observed among bedside nurses (non-managerial roles), with an average score that is 0.16 points higher than that of nurses holding managerial positions (such as head nurse of a clinic or department, head of a department or team). Despite this observed difference, the study reports no statistically significant disparity between these groups (t = 0.92; *p* = 0.357). In contrast, the perceived importance of professional development is noted to be more pronounced among nurses in managerial roles, surpassing their non-managerial counterparts by 0.41 points. Limiting factors affecting CPD participation appear less frequently among managerial nurses, with a mean score of 3.39 and a standard deviation of 1.02. This score is 0.72 points lower than that observed among bedside nurses. This difference is statistically significant (t = 2.86; *p* = 0.005), indicating distinct variances in constraints faced by nurses based on their roles within the organizational hierarchy. Furthermore, engagement in CPD activities shows a slightly higher prevalence among managerial nurses, with a mean score of 3.38 and a standard deviation of 0.78, which is 0.17 points higher than that of their non-managerial peers. However, this difference did not reach statistical significance (F = 0.63; *p* = 0.533), as detailed in Table 3.

The study reveals that participation in CPD activities does not exhibit significant variations in relation to the work schedule of nurses. This finding is supported by statistical analyses showing t-values of 0.27 (*p* = 0.844) and 0.50 (*p* = 0.685) for participation in CPD activities and activities related to CPD, respectively. Notably, the study highlights that the average level of importance attributed to personal professional development peaks among nurses working full-time (40 h per week, encompassing morning and afternoon shifts). This group’s importance level is 1.05 points higher than that of their part-time counterparts (those working less than 40 h per week). A statistically significant difference is observed in this context, with an F-value of 2.88 (*p* = 0.038), indicating a distinct disparity in how CPD is valued based on the number of working hours. The research identifies the highest average level of limiting factors in CPD among participants employed for 40–50 h per week (full-time—shift work). This level is 0.99 points higher compared to those working 50 h or more per week (full-time with on-call duties), among whom the lowest level of limiting factors is reported. A statistically significant difference in this regard is found (F = 5.56; *p* = 0.001), as detailed in Table 4.

After conducting the study, no statistically significant difference was found in participation in continuous professional development activities (t = 0.22; *p* = 0.829), the importance for personal professional development (t = 1.08; *p* = 0.281), and limiting factors in continuous professional development (t = 0.27; *p* = 0.784). However, the study identified a statistically significant difference in activities related to continuous professional development (t = 2.12; *p* = 0.036) among employees of surgical and intensive care units compared to other departments, where a higher level of engagement was present among employees of other departments (Table 5).

In the article, only those sections of the questionnaire that are relevant to the topic of the article are presented.

## 4. Discussion

Our study on the attitudes of nurses towards CPD reveals significant insights into the factors influencing their participation and motivation. The average level of engagement in CPD activities was 4.27 (±0.63), indicating a generally positive attitude towards CPD. This finding reflects the importance of CPD in maintaining high standards of patient care and adapting to evolving medical knowledge, as emphasized by Dale-Tam and Posner [15]. The systematic literature review on nurses’ CPD underscores its crucial role in nursing practice, highlighting its relevance to nurses’ roles and the enhancement of professional competence. Nurses are intrinsically motivated by personal and professional growth and extrinsically by organizational support. The review also points to the diverse needs of nurses in CPD, influenced by factors such as age and position [1].

Notably, higher participation in CPD activities was observed among nurses without managerial roles, contrary to expectations. This suggests that frontline nurses may perceive a more direct or practical benefit from CPD [16]. Conversely, the level of importance placed on professional development was significantly higher among nurses with managerial roles. This indicates a recognition of the value of CPD for leadership roles in nursing, akin to the findings by Curtis and Martin regarding the development of online CPD toolkits for nursing leadership [17].

The study also reveals that nurses in managerial positions encounter fewer barriers to engagement in CPD, with an average score of 3.39 (SD = 1.02). Rahmah and colleagues highlight the critical role of CPD in preparing nurses for challenging scenarios, noting that CPD tends to be sidelined when clinical demands peak. Therefore, integrating CPD into nursing practice is crucial for improving the quality of care, especially during high-stress periods like pandemics [18].

It was observed that there are no significant differences in CPD participation in relation to work schedules, but nurses employed full-time showed an increased perception of the importance of personal professional development. This finding mirrors the research by Mustapa and colleagues, exploring the enablers and barriers of CPD participation among nurses and midwives, emphasizing factors such as organizational support and personal motivations [19]. The correlation between full-time employment and increased prioritization of personal professional development, as revealed in our study, suggests that full-time nurses may have greater motivation or opportunities for engagement in CPD activities.

The study revealed a statistically significant disparity in CPD engagement (t = 2.12; *p* = 0.036) between staff in surgical and intensive care units compared to other departments. This suggests that the specific demands and constraints of working in intensive care and surgical settings may limit nurses’ opportunities to participate in CPD activities, particularly during regular work hours. In contrast, a Brazilian study explored the efficacy of a virtual learning environment, EDUCATE, in a pediatric cardiac intensive care unit. This study demonstrated that targeted virtual CPD activities significantly enhanced the theoretical knowledge of nurses. The success of this program was further supported by positive feedback and a strong interest in technological learning tools among participants [20]. This indicates that tailored CPD approaches, such as virtual learning environments, could potentially address the challenges faced by nurses in intensive and surgical units, enabling more effective and accessible professional development.

These findings resonate with the perspective of Skees, who emphasized the necessity for nurses to embrace lifelong learning to enhance the quality of care. Skees underscored the importance of technical skills and critical thinking, advocating for a work environment that values and promotes continuing education, thereby laying a foundation for excellence in nursing practice [21]. These findings contribute to a more nuanced understanding of the factors influencing nurses’ commitment to CPD, underscoring the importance of tailoring CPD opportunities to different roles and work contexts within the nursing profession. This ensures that all nurses, regardless of their position or schedule, have equitable access to professional development opportunities that meet their specific needs and circumstances.

These findings highlight the importance of understanding how various factors like workplace function, form of work, and the specificity of the workplace, influence professional development within the nursing sector. Therefore, these insights emphasize a critical aspect of nursing workforce development: the need to tailor CPD initiatives to maximize participation across different employment statuses. This approach recognizes that nurses, depending on their roles and working hours, face unique challenges and opportunities for professional growth.

### 4.1. Implications for Nursing Practice

The findings of this study underscore the critical role of dynamic CPD programs in the healthcare sector. While our study reiterates some fundamental points introduced earlier, it goes further to propose actionable strategies for nursing practice. Recognizing the positive attitude of nurses towards CPD, it becomes imperative for healthcare organizations to not only continue but also enhance their CPD offerings. These programs should be strategically aligned with the unique needs and career objectives of individual nurses. By doing so, healthcare institutions can ensure that CPD programs are not only accessible but are also genuinely beneficial, fostering a culture of continuous learning and professional excellence. This approach is pivotal in enhancing the quality of patient care, increasing job satisfaction among nurses, and potentially improving retention rates. Thus, the emphasis shifts from the mere availability of CPD to the quality and relevance of these programs, ensuring they are comprehensively beneficial to the nursing fraternity and the healthcare system at large.

### 4.2. Limitations of the Study

This study acknowledges certain limitations. It primarily focuses on nurses from a single healthcare institution, which may limit the generalizability of the findings to other settings or regions—while the results affirm the significance of CPD, it is pertinent to note that they are specific to the research conditions and the context of nurses at a single hospital in Split, Croatia. Additionally, the use of self-reported data might introduce response bias, potentially influencing the accuracy of the reported attitudes and motivations toward CPD.

## 5. Conclusions

This study highlights the importance of CPD among nurses, showing varying levels of engagement across different hospital departments. The research, conducted at the University Hospital Centre Split, found a generally positive attitude towards CPD activities. The study notably reveals that staff from non-surgical and non-intensive care units exhibit higher involvement in CPD activities compared to those in surgical and intensive care units. Nurses in managerial positions place greater importance on CPD for their career progression. Full-time nurses also have a stronger perception of the value of personal professional development than part-time nurses.

The study underscores the necessity to understand how different job conditions and roles within hospitals influence CPD participation and its perceived value. Targeted CPD programs to meet the specific needs of various departments, workplaces, and work functions could enhance active involvement and professional growth in nursing, crucial for improving healthcare services.

## Figures and Tables

**Table 1:** Demographic characteristics of the participants.

Age	
20–24 yrs old	20
25–29	48
30–34	16
35–39	30
40–44	19
45–49	7
50–54	5
55–59	4
60–65	2
Gender	
Female	138
Male	13
Work experience	
0–5	25
5–10	40
10–15	22
15–20	27
20–25	19
25–30	7
30–35	5
35–40	3
40–45	3

**Table 2:** Nurses’ engagement in CPD activities.

I Take Part in CPD Activities:	1		2		3		4		5 *		AS	SD
	n	%	n	%	n	%	n	%	n	%		
... in order to meet the requirements for registration in the future	3	1.99	3	1.99	12	7.95	44	29.14	89	58.94	4.41	0.87
... in order to increase my chances of promotion	13	8.61	8	5.3	24	15.89	35	23.18	71	47.02	3.95	1.27
... because further professional development is important to me	2	1.32	1	0.66	18	11.92	45	29.8	85	56.29	4.39	0.82
... to increase my professional status	3	1.99	6	3.97	20	13.25	41	27.15	81	53.64	4.26	0.97
... to improve my current qualifications	2	1.32	3	1.99	14	9.27	46	30.46	86	56.95	4.40	0.84
... because I consider it important to increase the status of my profession	3	1.99	4	2.65	13	8.61	47	31.13	84	55.63	4.36	0.90
... to support my career	4	2.65	1	0.66	18	11.92	49	32.45	79	52.32	4.31	0.90
... in order to carry out my work better	1	0.66	1	0.66	9	5.96	39	25.83	101	66.89	4.58	0.70
... in order to meet the requirements of the organization I work for	6	3.97	3	1.99	30	19.87	47	31.13	65	43.05	4.07	1.03
... in order to increase the quality of healthcare	3	1.99	1	0.66	21	13.91	50	33.11	76	50.33	4.29	0.88
... to prove to my employer that I am professionally competent	9	5.96	6	3.97	23	15.23	52	34.44	61	40.4	3.99	1.12
... because this is considered highly important in my professional environment	1	0.66	8	5.3	24	15.89	59	39.07	59	39.07	4.11	0.90
... in order to achieve a higher level of training	1	0.66	6	3.97	16	10.6	40	26.49	88	58.28	4.38	0.88
... in order to make a positive contribution to nursing practice	0	0	2	1.32	16	10.6	49	32.45	84	55.63	4.42	0.73
... to support my career potential/choice	3	1.99	3	1.99	17	11.26	43	28.48	85	56.29	4.35	0.90
... to improve my leadership abilities	4	2.65	6	3.97	18	11.92	42	27.81	81	53.64	4.26	1.00
Nurses’ engagement in CPD activities											4.27	0.63

* 1. Mainly disagree, 2. Partly disagree, 3. Neither agree nor disagree, 4. Partly agree, 5. Mainly agree.

**Table 3:** Correlation of participation in CPD activities with workplace function.

	With Managerial Role		Without Managerial Role		t	*p*
	AS	SD	AS	SD		
Participation in CPD activities	4.12	0.80	4.28	0.61	0.92	0.357
Important for own professional development.	4.47	0.54	4.06	0.52	2.77	0.006
Limiting factors of CPD	3.39	1.02	4.11	0.88	2.86	0.005
Activities related to CPD	3.38	0.78	3.21	0.96	0.63	0.533

t = test value of *t*-test, AS = arithmetic mean, SD = standard deviation.

**Table 4:** Correlation of participation in CPD activities with the form of work.

Form of Work	N	AS	SD	F	*p*
Participation in CPD activities					
40–50 h (full-time—shift work)	123	4.28	0.61	0.27	0.844
40 h (full-time—morning and afternoon shifts)	19	4.24	0.77		
50 h and more (full-time with on-call duties)	7	4.08	0.67		
Less than 40 h (part-time work)	2	4.12	0.00		
Important for personal and professional development					
40–50 h (full-time—shift work)	123	4.08	0.50	2.88	0.038
40 h (full-time—morning and afternoon shifts)	19	4.25	0.67		
50 h and more (full-time with on-call duties)	7	4.30	0.55		
Less than 40 h (part-time work)	2	3.20	0.55		
Limiting factors in continuous professional development					
40–50 h (full-time—shift work)	123	4.17	0.81	5.56	0.001
40 h (full-time—morning and afternoon shifts)	19	3.51	1.09		
50 h and more (full-time with on-call duties)	7	3.18	1.28		
Less than 40 h (part-time work)	2	3.88	1.58		
Activities related to continuous professional development					
40–50 h (full-time—shift work)	123	3.27	0.96	0.50	0.685
40 h (full-time—morning and afternoon shifts)	19	3.00	0.72		
50 h and more (full-time with on-call duties)	7	3.24	1.27		
Less than 40 h (part-time work)	2	3.00	0.86		

F = test value of ANOVA test, AS = arithmetic mean, SD = standard deviation

**Table 5:** Correlation of participation in continuing professional development activities with workplace.

	Surgical Departments and Intensive Care Units		Other Departments		t	*p*
	AS	SD	AS	SD		
Participation in CPD activities	4.25	0.58	4.27	0.65	0.22	0.829
Important for own professional development.	4.04	0.57	4.14	0.51	1.08	0.281
Limiting factors of CPD	4.07	0.84	4.02	0.96	0.27	0.784
Activities related to CPD	3.01	0.99	3.35	0.90	2.12	0.036

t = test value of *t*-test, AS = arithmetic mean, SD = standard deviation.

## Data Availability

Study data are available upon request from the corresponding author.
